# Glucagon-like peptide-1 receptor regulates endoplasmic reticulum stress-induced apoptosis and the associated inflammatory response in chondrocytes and the progression of osteoarthritis in rat

**DOI:** 10.1038/s41419-017-0217-y

**Published:** 2018-02-12

**Authors:** Jian Chen, Jun-Jun Xie, Ke-Si Shi, Yun-Tao Gu, Cong-Cong Wu, Jun Xuan, Yue Ren, Long Chen, Yao-Sen Wu, Xiao-Lei Zhang, Jian Xiao, De-Zhong Wang, Xiang-Yang Wang

**Affiliations:** 1 Department of Orthopaedics, The Second Affiliated Hospital and Yuying Children’s Hospital of Wenzhou Medical University, Wenzhou, 325027 People’s Republic of China; 2 School of Pharmaceutical Sciences, Key Laboratory of Biotechnology and Pharmaceutical Engineering, Wenzhou Medical University, Wenzhou, 325027 Zhejiang China; 3 The first clinical college, Wenzhou Medical University, 325027 Wenzhou, People’s Republic of China; 4 Biological science academy, Wenzhou university, 325027 Wenzhou, Zhejiang China

## Abstract

Treatments for osteoarthritis (OA) are designed to restore chondrocyte function and inhibit cell apoptosis. Previous studies have shown that activation of the glucagon-like peptide-1 receptor (GLP-1R) leads to anti-inflammatory and anti-apoptotic effects. However, the role of GLP-1R in the pathological process of OA is unclear. In present work, we aimed to demonstrate the potential effect of GLP-1R on chondrocytes and elucidate its underlying mechanisms. We found that activation of GLP-1R with liraglutide could protect chondrocytes against endoplasmic reticulum stress and apoptosis induced by interleukin (IL)-1β or triglycerides (TGs). These effects were partially attenuated by GLP-1R small interfering RNA treatment. Moreover, inhibiting PI3K/Akt signaling abolished the protective effects of GLP-1R by increase the apoptosis activity and ER stress. Activating GLP-1R suppressed the nuclear factor kappa-B pathway, decreased the release of inflammatory mediators (IL-6, tumor necrosis factor α), and reduced matrix catabolism in TG-treated chondrocytes; these effects were abolished by GLP-1R knockdown. In the end, liraglutide attenuated rat cartilage degeneration in an OA model of knee joints in vivo. Our results indicate that GLP-1R is a therapeutic target for the treatment of OA, and that liraglutide could be a therapeutic candidate for this clinical application.

## Introduction

Osteoarthritis (OA) is a prevalent progressive and degenerative joint disease that results in personal activity limitations, disability, and great economic burden worldwide^1^. Various factors contribute to the initiation and progression of OA, such as age, gender, body weight, heredity, and mechanical injury^2,3^. The main treatment for OA has been to delay its development; few anti-inflammatory therapies have been used to relieve the symptoms of OA. Therefore, fully understanding the pathogenesis of OA would be helpful in exploring novel therapies.

Several studies have demonstrated inflammation-related changes in OA cartilage. The expression of pro-inflammatory mediators, including interleukin-1 (IL-1), IL-6, and tumor necrosis factorα (TNF-α), increase in cartilage, bone, and synovium, contributing to the initiation of OA^4,5^. These cytokines activate the nuclear factor kappa-B (NF-κB) signaling pathway,inducing the upregulation of apoptotic proteins and matrix-degrading enzymes, such as metalloproteinases (MMPs), which irreversibly degrade the extracellular matrix (ECM)^6,7^. A previous study showed that systematic blockade of IL-6 reduced expression of MMPs and ADAMTSs (a disintegrin and metalloproteinase with thrombospondin motifs) could alleviate medial meniscus-induced OA cartilage lesions^8^.

Endoplasmic reticulum (ER) stress is another critical etiology in the OA pathological process^9–11^. Accumulation of misfolded proteins in the ER triggers the unfolded protein response (UPR), in which C/EBP homologous protein (CHOP) is activated, subsequently activating the caspase family and inducing chondrocyte death^12^. ATF6, a regulator of ER stress, can augment XBP1S gene expression and modulate ER stress-mediated apoptosis via caspase-3 and caspase-12 in OA cartilage^13^. In addition, it has been reported that the pro-inflammatory factor IL-1β induces ER stress-related apoptosis in cultured chondrocytes^14^. Furthermore, IL-1β plays a critical role in the initiation of OA; evidence suggests that IL-1β can accelerate anabolic activities in chondrocytes^15^. In this study, IL-1β was used to induce ER stress and subsequent apoptosis.

Glucagon-like peptide-1 (GLP-1) is an incretin hormone, produced by enteroendocrine cells (L cells) to regulate glucose and energy homeostasis via GLP-1R binding^16^. Not only does this have functional implications for diabetes mellitus, but it also has potential protective effects in the cardiovascular and nervous systems. GLP-1 can decrease cardiomyocyte destruction and increase cell viability after ischemia–reperfusion injury^17^. Exendin-4, a GLP-1R agonist, significantly promoted locomotor recovery in rats after spinal cord injury via inducing autophagy and inhibiting apoptosis^18^. Importantly, recent studies showed that activation of GLP-1R inhibited ER stress in several models. GLP-1R agonizts prevented the onset of ER stress and subsequent apoptosis in human umbilical vein endothelial cells when stimulated by high glucose^19^. Additionally, a GLP-1R antagonist enhanced ER stress-related apoptosis in high glucose-stimulated HK-2 cells^20^.

However, to our knowledge, the role of GLP-1R in OA has not been reported to date. We hypothesized that the GLP-1R agonist, liraglutide, might alleviate chondrocyte apoptosis and ECM degradation by regulating ER stress. Moreover, activation of GLP-1R may be involved in ER stress-induced inflammatory responses. We also analysed the protective effect of liraglutide on cartilage degeneration in vivo. These new insights suggest that GLP-1R may be a potential target for the development of novel drugs in the treatment of OA.

## Results

### GLP-1R is mainly expressed in cartilage chondrocytes and decreases in degenerative cartilage

**Fig. 1:**
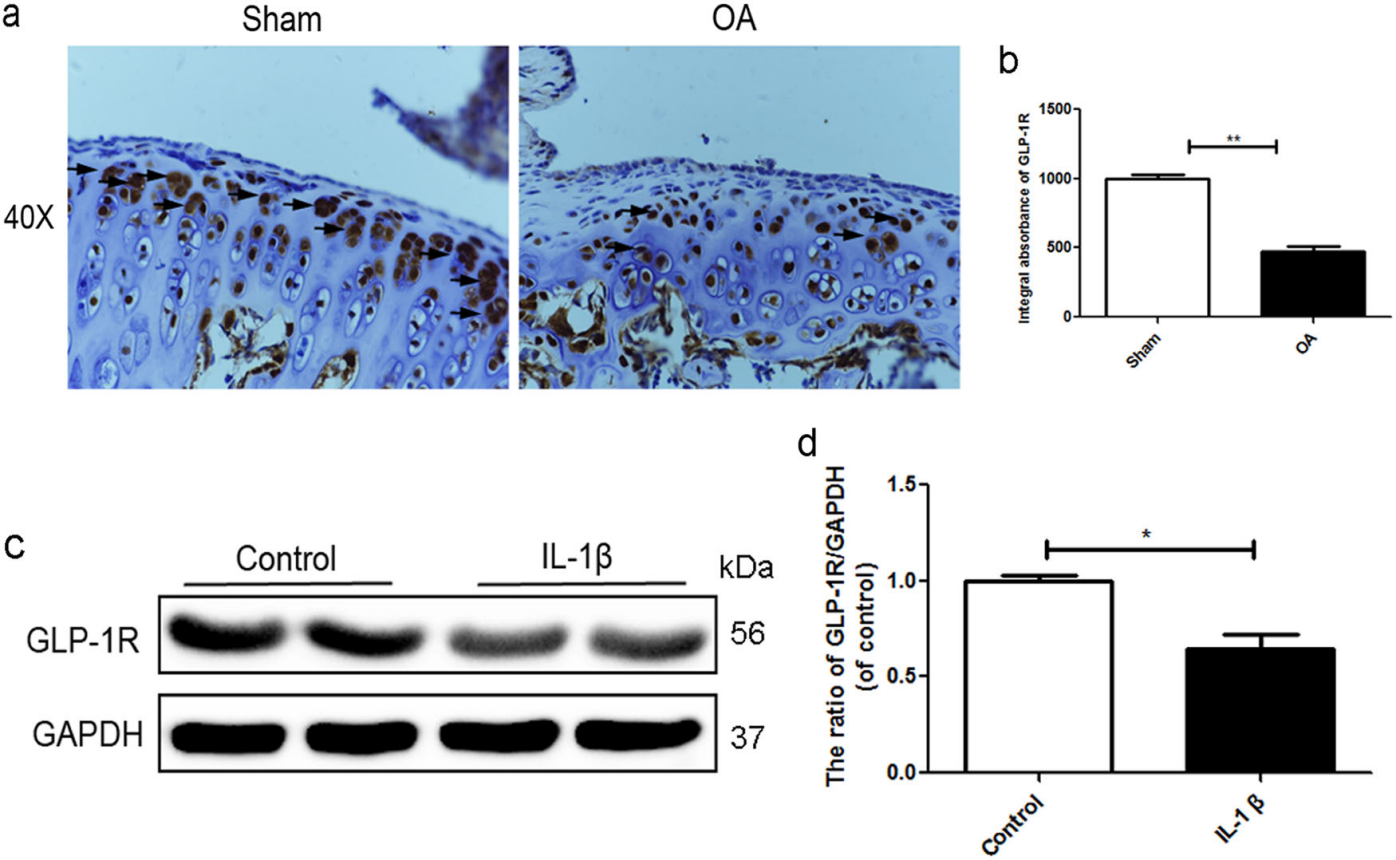
GLP-1R is mainly expressed in cartilage chondrocytes and decreases in degenerative cartilage. **a**, **b** Immunohistochemical staining of GLP-1R expression in normal and OA group (40×). **c**, **d** Representative western blots and quantification data of GLP-1R in normal and OA group. Columns represent mean ± SD, Significant differences between the normal and OA group are indicated as *P < 0.05, *n* = 5

Few articles have reported the role of GLP-1R in the articular cartilage degeneration. As shown in Fig. 1a, b, immunohistochemical staining and corresponding quantification of GLP-1R showed that GLP-1R of membrane is slightly but significantly downregulated in degenerative cartilage compared with normal cartilage. We used IL1-β to mimic OA in chondrocytes in vitro. Western blotting data demonstrated that the expression of GLP-1R was lower in response to IL-1β stimulation than control group (Fig. 1c, d). Together, these results suggested a potential role of GLP-1R in OA.

### Activation of GLP-1R by liraglutide decreases apoptosis in chondrocytes

**Fig. 2:**
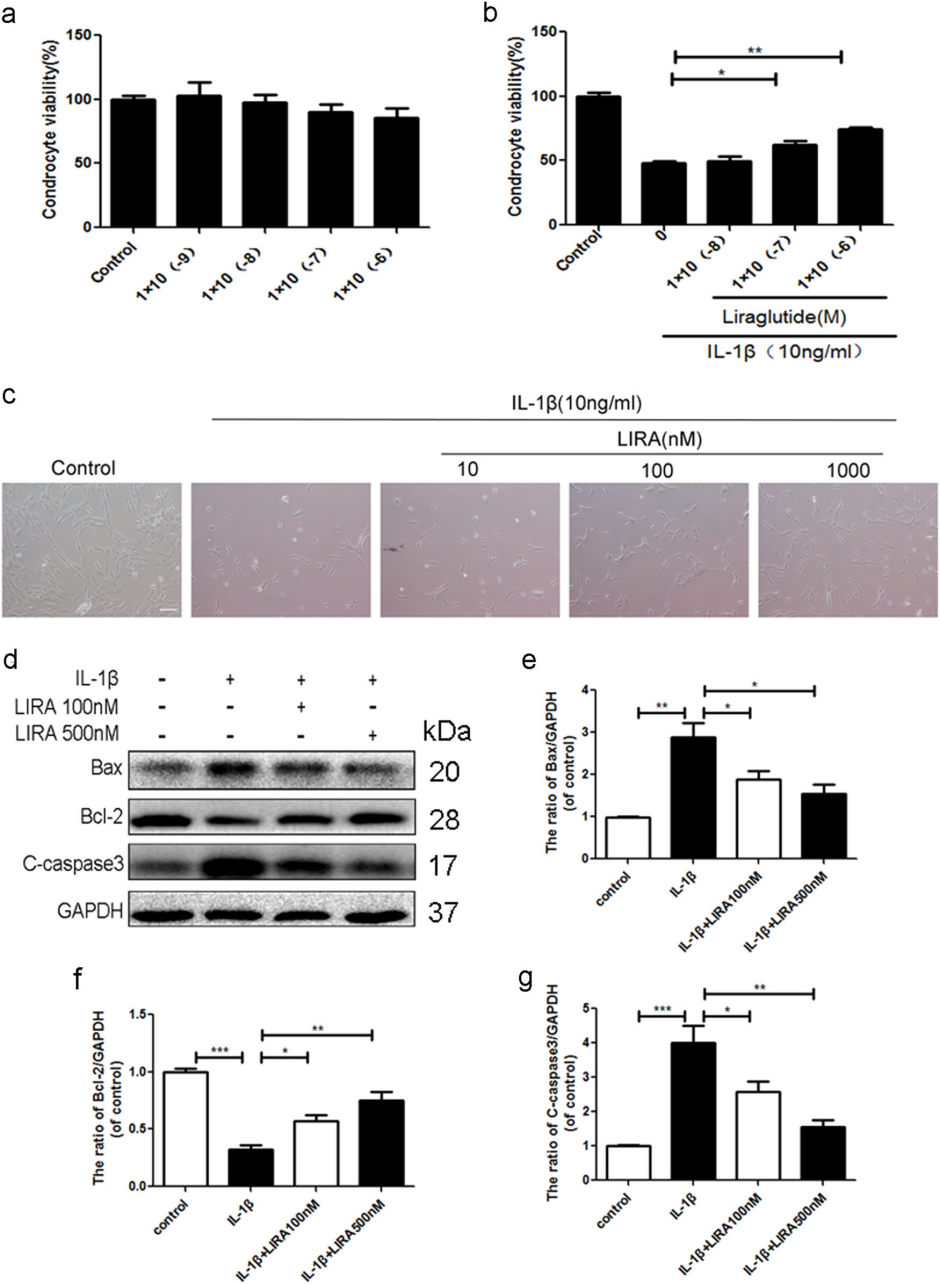
Activation of GLP-1R by liraglutide decreases apoptosis in chondrocytes. **a** CCK-8 assays of chondrocytes treated with various concentrations of liraglutide for 24 h as shown above. **b** CCK-8 assays of liragltide-pretreated chondrocytes stimulated by IL-1β. **c** Chondrocytes were pretreated with liraglutide and then IL-1β and imaged by phase-contrast microscopy (20×). **d**–**g** Representative western blots and quantification data of Bax, Bcl-2 and cleaved-caspase 3 in each group. Columns represent mean ± SD, Significant differences between the treatment and control groups are indicated as *P < 0.05, **P < 0.01, ***P < 0.001, *n* = 5

To assess the role of GLP-1R in chondrocytes, we used liraglutide, a GLP-1R agonist, to activate GLP-1R with various concentrations. As shown in Fig. 2a, liraglutide exerted no significant cytotoxic effect on chondrocytes. And we noted that liraglutide concentrations up to 10^−7^ M showed significant protective effect on IL-1β treated-chondrocytes (Fig. 2b). Chondrocytes shrunk in size, and reduced in number when exposed to IL-1β, which was partially reversed by liraglutide (Fig. 2c). The western blotting analysis also showed that activation of GLP-1R significantly decreased the level of pro-apoptotic protein cleaved-caspase3 and Bax and increased the level of anti-apoptotic protein Bcl-2, compared with IL-1β induced-chondrocytes (Fig. 2d–g). Take together, these results showed that GLP-1R may exert an anti-apoptotic effect on chondrocytes.

### The anti-apoptotic effects of GLP-1R was modulated by PI3K/Akt signaling

**Fig. 3:**
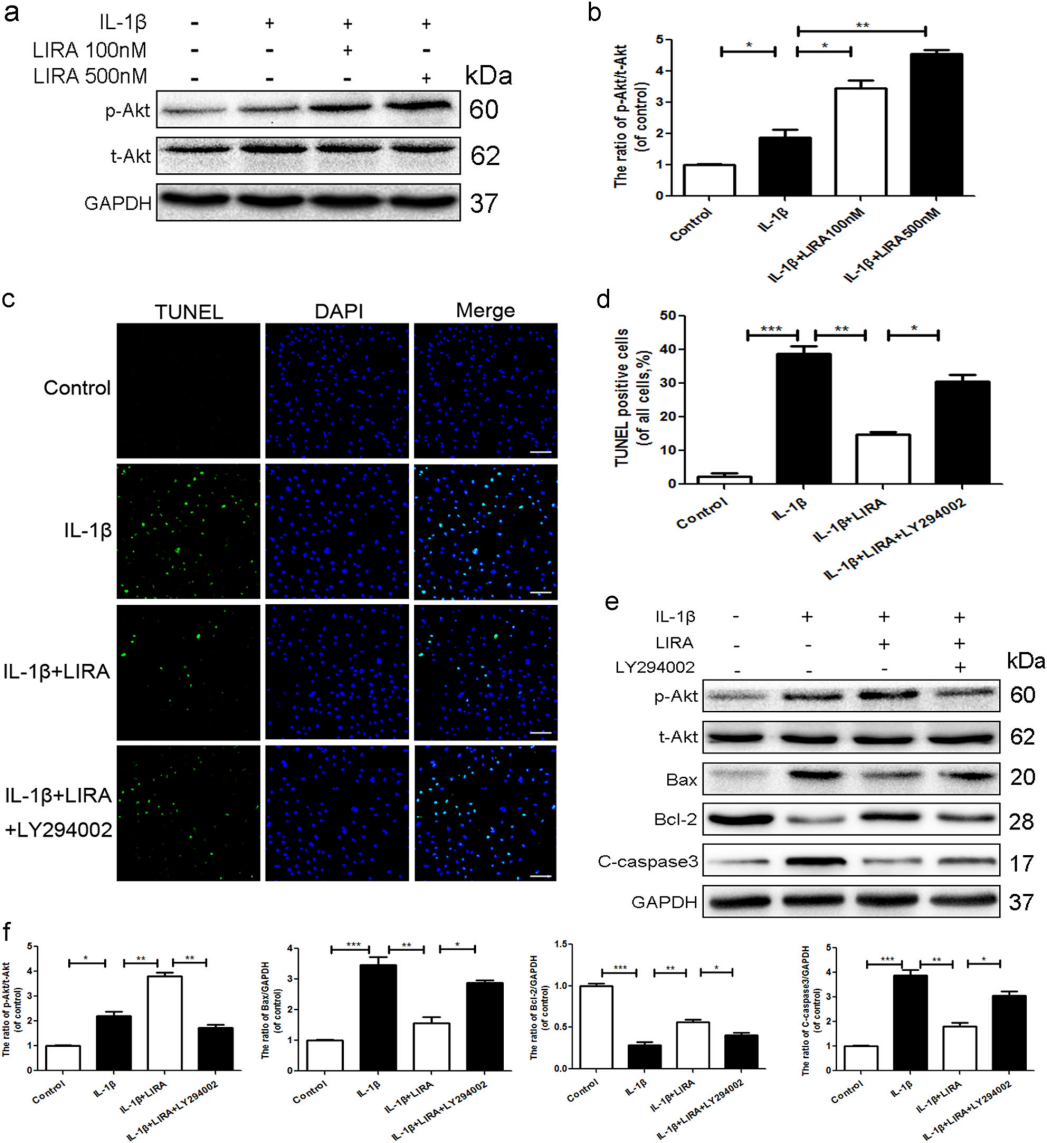
The anti-apoptotic effects of GLP-1R was modulated by PI3K/Akt signaling in chondrocytes. **a**, **b** Representative western blots and quantification data of p-Akt and Akt in each group. **c**, **d** TUNEL assay was used to assess the apoptosis of each group (scale bar: 50 μm). **e**, **f** Representative western blots and quantification data of p-Akt, Akt, Bax, Bcl-2, and cleaved-caspase 3 in each group. Columns represent mean ± SD, Significant differences between the treatment and control groups are indicated as *P < 0.05, **P < 0.01, ***P < 0.001, *n* = 5

GLP-1 analogs regulate various biological processes by activating GLP-1R/PI3K/Akt signaling^21,22^. Interestingly, Western blotting demonstrated that GLP-1R activation further upregulated the ratio of p-Akt/t-Akt when compared to IL-1β group (Fig. 3a, b). To explore whether the protective effects of GLP-1R were modulated by PI3K/Akt signal pathway, LY294002, a specific PI3K inhibitor was used to treat chondrocytes combined with liraglutide treatment. TUNEL staining showed that IL-1β markedly enhanced the apoptotic level, when compared to the control group. And GLP-1R activation significantly decreased apoptotic activity, which were reversed partially by LY294002 (Fig. 3c, d). Furthermore, Western blot assays noted increasing expression of apoptotic proteins active caspase 3 and Bax in the IL-1β group, which was abolished by liraglutide. Conversely, liraglutide upregulated the expression of anti-apoptotic marker Bcl-2 compare to IL-1β group. Furthemore, LY294002 abolished GLP-1R’s anti-apoptotic effect (Fig. 3c–e). To further demonstrate the relationship between GLP-1R and PI3K/Akt signaling, GLP-1R siRNA was used to treat chondrocytes. And GLP-1R knockdown markedly abolished the effect of liraglutde to modulate the ratio of p-Akt/t-Akt after liraglutide treatment, when compared with both the control and negative control siRNA group (Supplementary Figure S1a and c). Together, these results demonstrated that PI3K/Akt signaling was involved in the protective effect of GLP-1R.

### Activation of GLP-1R inhibits ER stress and associated apoptosis via activation of PI3K/Akt signaling in chondrocytes

**Fig. 4:**
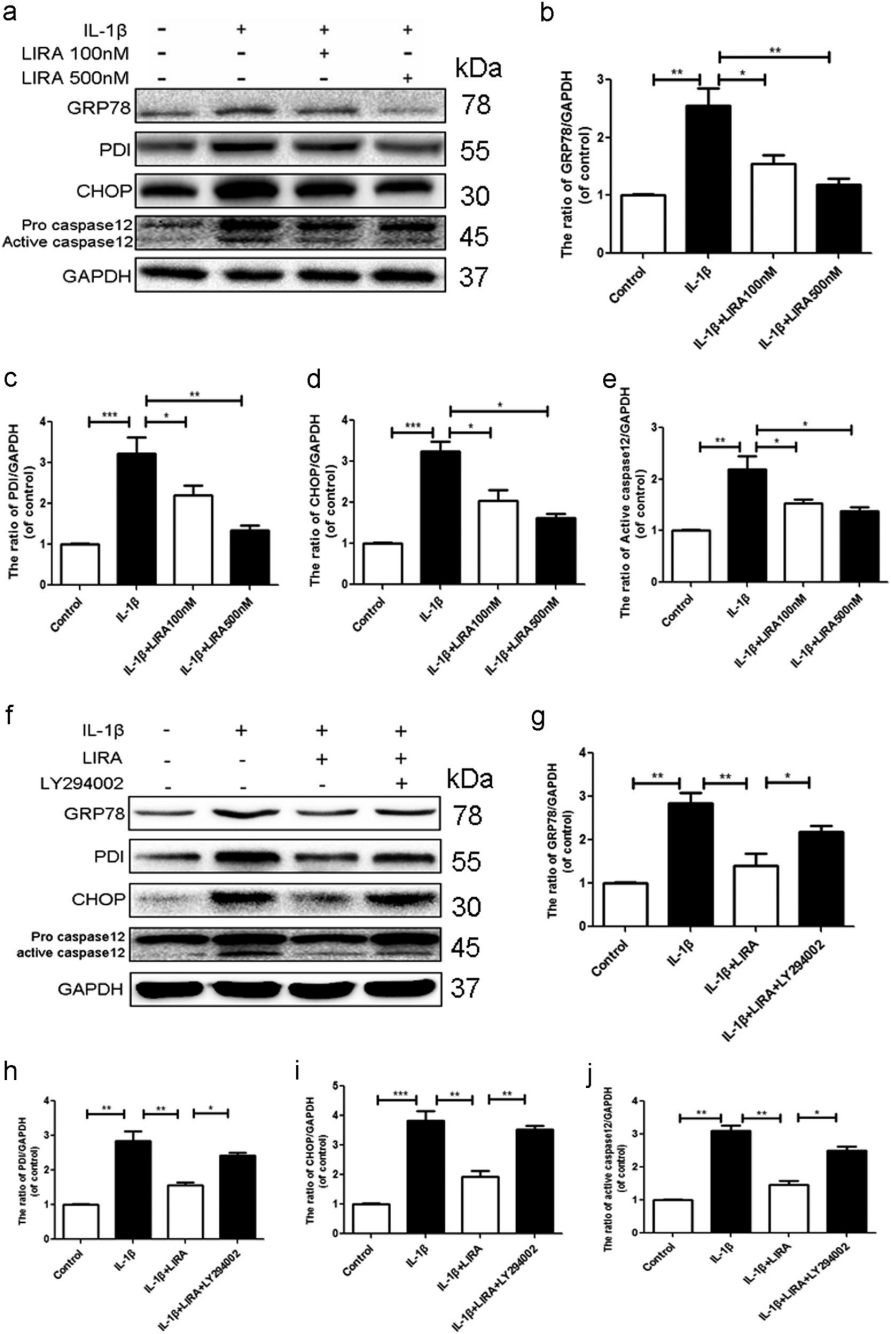
Activation of GLP-1R inhibits ER stress via activation of PI3K/Akt signaling in chondrocytes. **a**–**e** Representative western blots and quantification data of GRP78, PDI, caspase12 and CHOP in each group. **f**–**j** Representative western blots and quantification data of GRP78, PDI, caspase12 and CHOP in each group. Columns represent mean ± SD, Significant differences between the treatment and control groups are indicated as *P < 0.05, **P < 0.01, ***P < 0.001, *n* = 5

To test whether ER stress was related to the anti-apoptotic effect of GLP-1R, the GRP78, PDI, caspase12 and CHOP, which regarded as the markers of ER stress, were assessed by western blotting. As shown in Fig. 4a–e, IL-1β significantly increased the ER stress related protein. However, activation of GLP-1R by liraglutide attenuated the IL-1β induced-upregulation of ER stress-related proteins in chondrocytes. These results indicated that the protective role of GLP-1R may involve the inhibition of ER stress-related protein.

To further clarify the specific mechanism of GLP-1R in chondrocytes, we used the LY294002 to treat chondrocytes with liraglutide. Similarly, inhibition of PI3K/Akt signaling abolished the protective effect by liraglutide, indicating that PI3K/Akt signaling was involved in the GLP-1R’ effects of inhibition of ER stress (Fig. 4f–j). To further demonstrate whether GLP-1R was associated with ER stress in cartilage, we used TG, a classic ER stress inducer, to cause ER stress and GLP-1R siRNA was used to knockdown the GLP-1R in chondrocytes. And western bolt results noted that activation of GLP-1R inhibited the TG-induced the increase of the expression of GRP78, PDI, CHOP and active caspase12 compared to control group, which were partially reversed by GLP-1R siRNA (Supplementary Figure S2a–e). In order to further demonstrate that ER stress is involved in the anti-apoptotic effect of GLP-1R, TUNEL assay was performed to assess the level of apoptotic cells. Compared with the TG treatment group, activation of GLP-1R by liraglutide markedly decreased the number of apoptotic cells. However, GLP-1R knockdown greatly reduced anti-apoptotic activity of liraglutide (Supplementary Figure S3a and b). Moreover, the western blot results showed treatment of activating GLP-1R greatly inhibited the increase of Bax and cleaved caspase3 and the decrease of Bcl-2 induced by TG. However, GLP-1R siRNA weakened the effect of liraglutide (Supplementary Figure S3c–f). These results further proved that GLP-1R activation contributes to the inhibition of ER stress and subsequent apoptosis resulted from GLP-1R activation.

### GLP-1R may relate ER stress and NF-κB pathway in chondrocytes

**Fig. 5:**
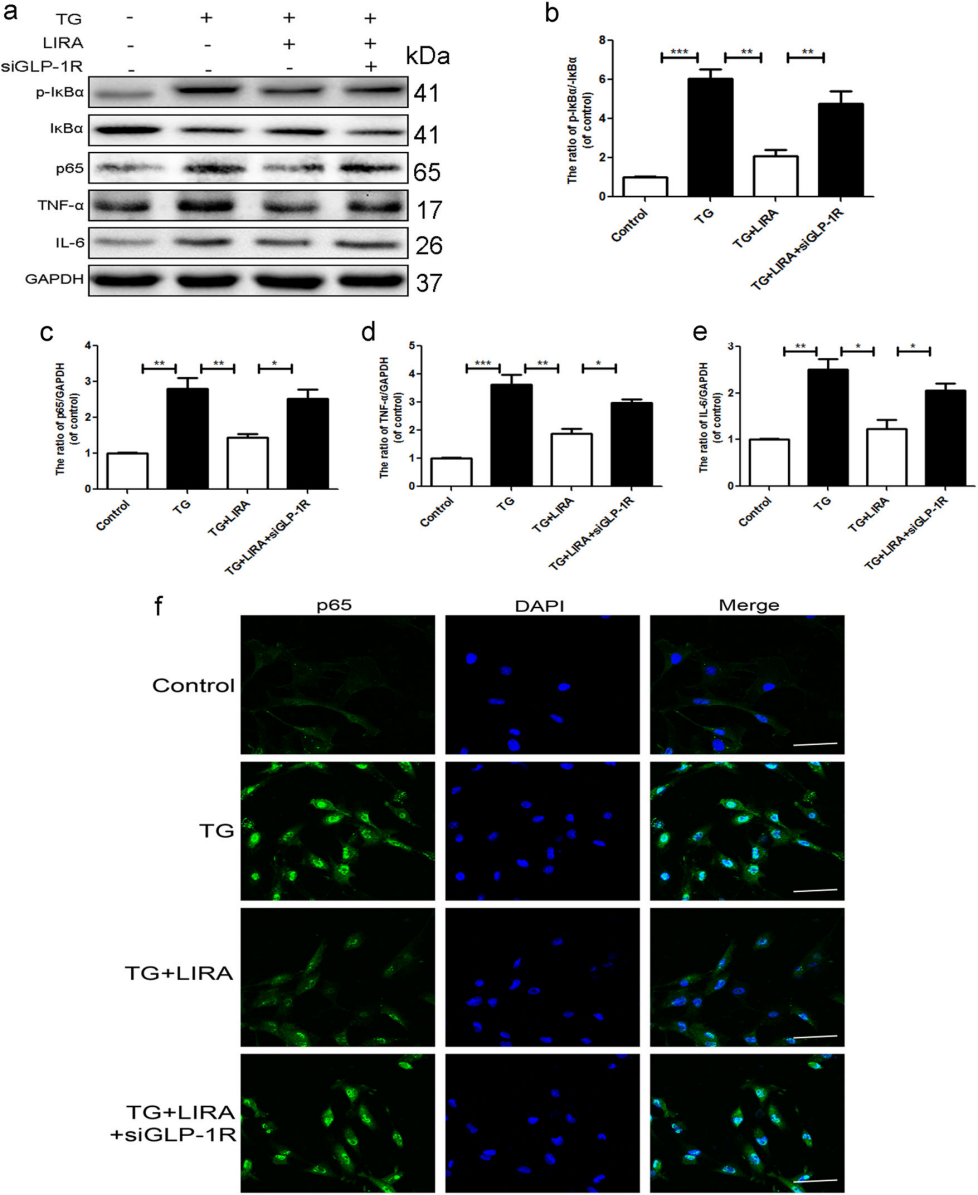
GLP-1R may relate ER stress and NF-κB pathway in chondrocytes. **a**–**f** Representative western blots and quantification data of p65, p-IκBα and IκBα, IL-6, TNF-α in each group. **g** Immunofluorescence staining of P65 proteins (green) and nucleus (blue) was labeled with DAPI (scale bar: 50 μm). Columns represent mean ± SD, Significant differences between the treatment and control groups are indicated as *P < 0.05, **P < 0.01, ***P < 0.001, *n* = 5

Several studies have noted the inflammation-related changes in the OA cartilage. Then we test the protein levels of p-IκBα, IκBα, and NF-κB (p65) under the TG treatment using the western blot. IκBα was an upstream target of NF-κB, its phosphorylation and degradation contribute to the activation of the NF-κB pathway. Compare to the control group, higher expression of p65, IL-6, TNF-α, and ratio of p-IκBα/IκBα were noted in TG group indicating the activation of NF-κB pathway and associated inflammatory response may relate to ER stress. However, liraglutide treatment inhibited the activation of NF-κB and subsequent inflammatory response, which was reversed by GLP-1R siRNA (Fig. 5a–e). NF-κB nuclear translocation triggers release of inflammatory mediators. Immunostaining assays showed that activating GLP-1R significantly attenuated TG induced nuclear translocation of NF-κB, which was also abolished by GLP-1R siRNA (Fig. 5f). Together, these results showed that GLP-1R may play an essential role in ER stress-induced inflammatory pathways.

### GLP-1R activation decreased the ECM catabolic activity in TG-treated chondrocytes

**Fig. 6:**
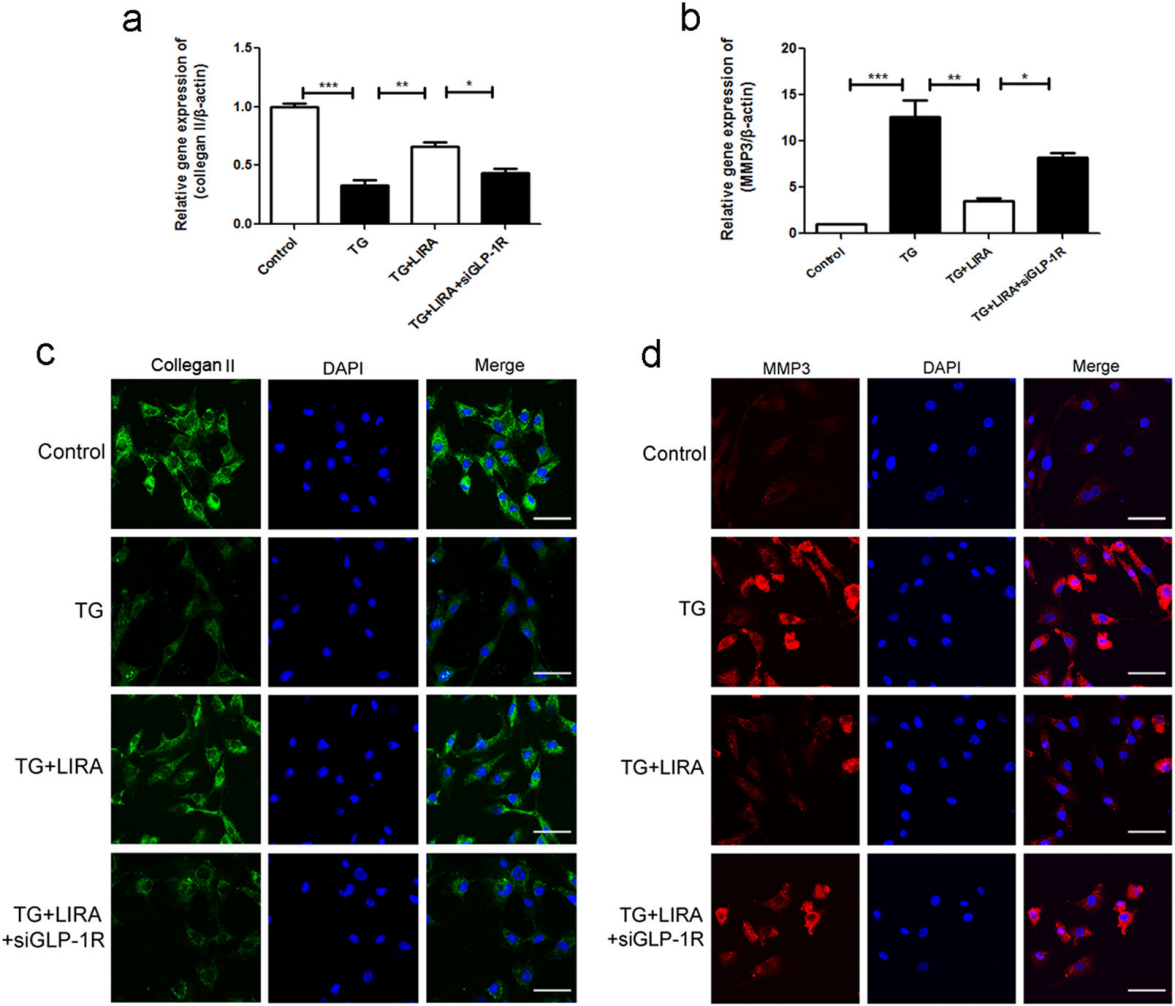
GLP-1R activation decreased the ECM catabolic activity in TG-treated chondrocytes. **a**, **b** The relative mRNA expression of MMP-3 and collegan-II in each group. **c**, **d** Immunofluorescence staining of MMP-3 and collegan-II proteins in each group (scale bar: 50 μm). Columns represent mean ± SD, Significant differences between the treatment and control groups are indicated as *P < 0.05, **P < 0.01, ***P < 0.001, *n* = 5

We tested the effect of liraglutide on TG-induced metabolic activity of ECM in chondrocytes using PCR and immunofluorescence assay, including major ECM protein collegan-II and ECM degrading protein MMP-3. As shown in Fig. 6a, b, TG treatment markedly reduced mRNA expression of collegan II, and upregulated the mRNA levels of MMP3. However, liraglutide attenuated the ECM catabolic activity, and the effect of GLP-1R were significantly abolished by GLP-1R siRNA. And immunofluorescence evaluation of collagen-II and MMP3 protein expression keeps consistent with the mRNA results (Fig. 6c, d), indicating GLP-1R may regulate the ECM metabolic activity in chondrocyte.

### Liraglutide treatment ameliorated chondrocytes apoptosis and cartilage degeneration in rat OA model

**Fig. 7:**
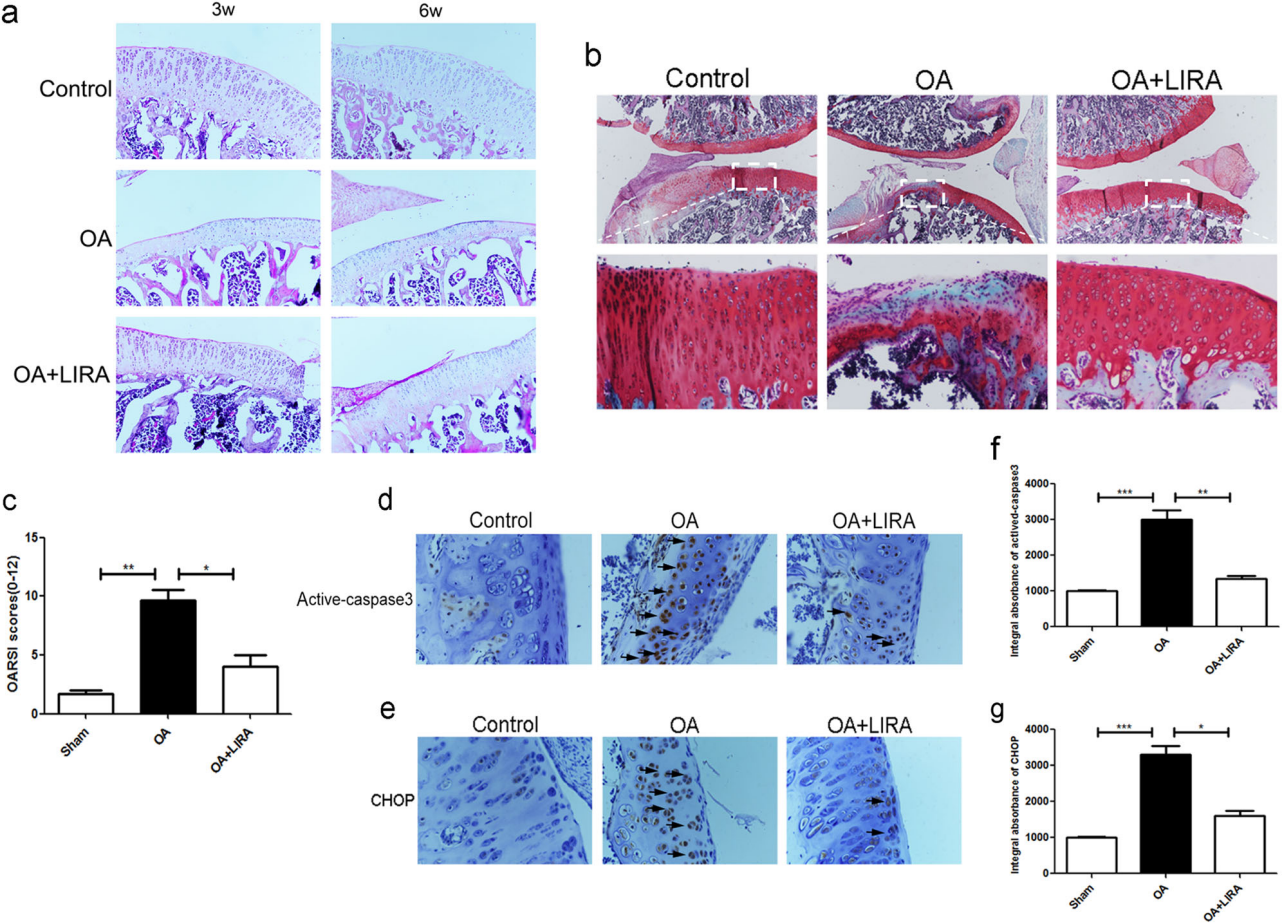
Liraglutide treatment ameliorated chondrocytes ER stress, associated apoptosis and cartilage degeneration in rat OA model. **a** HE staining in each group at 3 and 6 weeks (20×). **b** Safranin O staining in each group at 6 weeks (10×). **c** OARSI scores of each group at 6 weeks. **d**–**g** Immunohistochemical staining of cytoplasmic CHOP and cleaved-caspase3 expression in each group (40×)

The histopathological change of the matrix layer and articular structure was observed by HE, Safranin O staining and OARSI scores. In the OA group, morphological structure of articular cartilage showed significant destruction, including cartilage erosion, proteoglycan and cellular loss, compared with control group. Importantly treatment with liraglutide significantly reduced the severity of cartilage degeneration in the OA cartilages, which was consistent with OARSI scores (Fig. 7a–c). And immunohistochemical staining and corresponding quantification showed that liraglutide can greatly decreased the cytoplasmic CHOP and caspase3 positive in rat articular cartilage compared with OA model (Fig. 7d–g), which confirmed the results of our in vitro studies. Our data showed that GLP-1R agonist has a significant protective effect on vivo.

## Discussion

The process of OA has been attributed to several processes, such as aging and excessive mechanical loading, which trigger a series of molecular events leading to cell death and structural damage in articular cartilage, particularly inflammation^23–25^. The degradation of articular cartilage is mainly due to dysregulation of chondrocyte apoptosis and excessive catabolism of ECM^26–28^. Several studies reported that chondrocyte apoptosis contributes to cellular changes in cartilage and loss of articular cartilage^29,30^. Many important mediators, such as IL-1β and TNF-α, recruit death-inducing signaling complexes via binding with respective ligands, and subsequently activate the caspase signaling pathway, ultimately leading to internucleosomal DNA fragmentation^31,32^. Inflammation plays a vital role in ECM metabolic processes, and increasing evidence suggests that inflammation-mediated overexpression of MMPs results in a progressive matrix catabolic process^5,33^

Specific ligands bind to GLP-1R and subsequently mediate multiple biological processes, which occur in many tissues, such as the brain, heart, and pancreatic islets^34–37^. Nevertheless, little is known about the role of GLP-1R in OA. Increasing evidence indicates that GLP-1R has extensive links with anti-apoptotic effects. GLP-1 analogs reportedly protect cholangiocytes against apoptosis by preventing mitochondrial translocation of Bax and caspase-3 activation, in vitro and in vivo^38^. It has been demonstrated that the protective effects of GLP-1R could be mediated by PI3K/Akt signaling^21,22^. The PI3K/Akt pathway plays pivotal role in growth and survival of many biological processes, such as ischemia injury and degenerative diseases. Phosphorylated Akt reduces the pro-apoptotic protein Bad, and attenuates apoptotic activity after brain injury^39^. Akt also inactivates pro-caspase-9, and causes caspase9 dephosphorylation, decreasing the level of apoptosis^40^. It has been reported that GLP-1R activation inhibits hypoxia/reoxygenation damage in cardiac microvascular endothelial cells via the PI3K/Akt signaling^21^. This work first noted the decreasing expression of GLP-1R in degenerative cartilage, indicating the dysfunction of GLP-1R pathway during the process of OA. And to further explore whether the activation of GLP-1R exerted a protective effect on chondrocytes, the GLP-1R agonist liraglutide was used in IL-1β-stimulated chondrocytes. Activation of GLP-1R markedly reduced the levels of pro-apoptotic Bax and cleaved-caspase3, and increased anti-apoptotic Bcl-2 in IL-1β-induced chondrocytes, indicating that GLP-1R may be a potential target for the treatment of OA. To further explore the potential mechanism of GLP-1R, LY294002, a specific PI3K/Akt inhibitor was used to treat chondrocytes combined with liraglutide treatment. Interestingly, LY294002 abolished the anti-apoptotic effects of GLP-1R, indicating that PI3K/Akt signaling may involve in the protective effects of GLP-1R.

ER stress is closely related to apoptosis in OA. During the process of ER stress, UPR sensors separate from chaperone proteins, such as GRP78 and PDI, and then become phosphorylated, which activates CHOP and apoptotic proteins, such as caspase-12, to induce apoptosis^41^. Evidence shows that there is less apoptosis and cartilage degeneration in arthrosis of CHOP knockout mice^42^. Moreover, it has been shown that a GLP-1R agonist attenuated ER stress by downregulating ATF-4, a biomarker of ER stress, and inhibited apoptosis in INS-1 β-cells, which could improve cell survival^43^. Additionally, exenatide 4, a GLP-1R agonist, alleviated lipid-induced ER stress and improved hepatic steatosis in high-fat diet -fed mice via sirtuin 1 (SIRT1)^44^. Our data showed that pretreatment with a GLP-1R agonist alleviated the expression of ER stress-related proteins, such as PDI, GRP78, CHOP, and caspase-12, which increase in response to ER stress and are considered to be mediators of apoptotic pathways^45,46^, which were partial reversed by LY294002. These results indicated that the protective effect of the GLP-1R agonist may be attributable to the inhibition of ER stress in chondrocytes. And inhibiting PI3K/Akt pathway abolished the protective effects of GLP-1R by attenuating ER stress. Moreover, to confirm the protective role of agonist-activated GLP-1R, siRNA targeted to GLP-1R and TG, an inducer of ER stress, were used to treat chondrocytes. Treatment with liraglutide reduced the TG-induced accumulation of these ER stress-related proteins and subsequently decreased ER stress related-apoptosis in chondrocytes. These effects were abolished by GLP-1R siRNA treatment, further demonstrating that GLP-1R may exert a protective effect in chondrocytes against ER-associated apoptosis in OA. Additionally, in vivo experiments also demonstrated that treatment with liraglutide attenuated ER stress related-apoptosis and the destruction of articular cartilage compared with the OA group. These results indicated a therapeutic effect of the GLP-1R agonist in the rat OA model and were consistent with the results in vitro.

Inflammatory responses are a vital contributor to ECM degradation in OA^5,47^. Various inflammatory factors can trigger the phosphorylation and degradation of IκB and translocate NF-κB into the nucleus, facilitating inflammatory protein synthesis and pro-inflammatory molecule release (IL-6, TNF-α), leading to cartilage degeneration^48–50^. Previous studies showed that NF-κB (p65) knockdown could significantly reduce the expression of MMPs and inflammatory factors in chondrocytes that were treated with IL-1β and TNF-α^51^. Moreover, other studies reported that the GLP-1R signaling pathway is anti-inflammatory via regulation of NF-κB signaling and subsequent promotion of cellular function in different cells, including pancreatic β-cells, macrophages, and endothelial cells^52–55^.

Recent studies demonstrated that ER stress is involved in inflammatory responses^56–58^. ER-resident IRE1α is required to activate NF-κB translation in response to ER stress through TRAF2-mediated formation of IRE1α and IKK complex. Inhibition of NF-κB suppressed ER stress-induced cell death in MCF-7 cells^59^. Therefore, we assumed that the GLP-1R agonist inhibited ER stress and subsequently the inflammatory response during the process of ECM degradation. These results showed that TG-induced ER stress triggered nuclear translation of NF-κB, contributing to pro-inflammatory cytokine transcription and subsequently promoting an ECM catabolic process. Furthermore, activation of GLP-1R significantly inhibited the ER stress-induced nuclear translation of NF-κB and reduced expression levels of inflammatory molecules in TG-induced chondrocytes. Moreover, liraglutide prevented the degradation of ECM components by downregulating matrix-degrading enzymes, such as MMP-3, suggesting that the GLP-1R agonist modulated the process of reducing the ER stress-related inflammatory response and alleviated the ECM catabolic process. Moreover, the anti-inflammatory effect of liraglutide via regulating ER stress was abolished by GLP-1R siRNA.

**Fig. 8:**
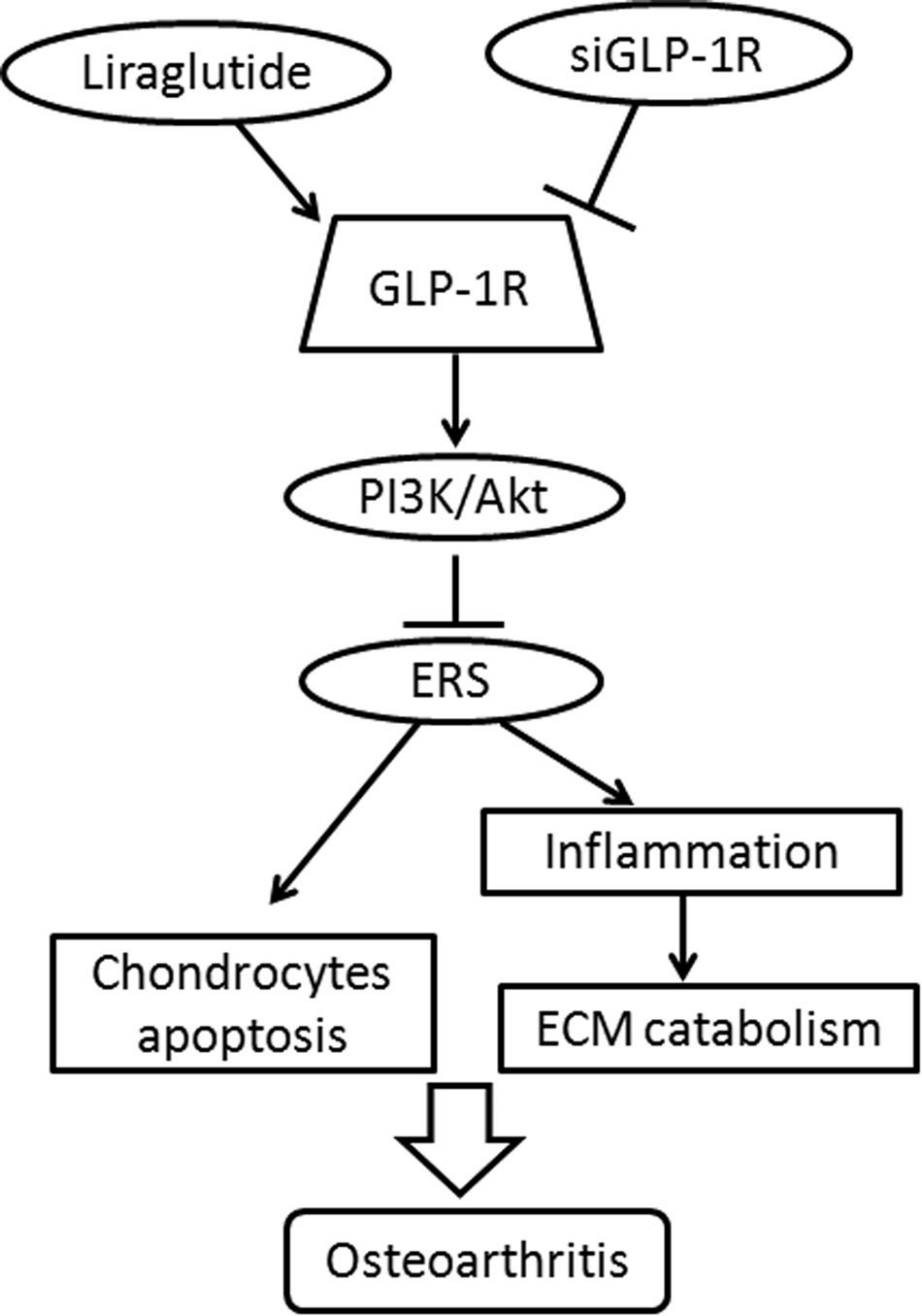
A schematic diagram depicting the potential molecular mechanisms underlying GLP-1R mediated-chondroprotection against apoptosis and inflammation *via* regulating PI3K/Akt/ER stress and NF-κB signaling

In conclusion, this study provides mechanistic evidence to demonstrate the anti-apoptotic and anti-inflammatory effects of GLP-1R activation in chondrocytes, in vivo and in vitro. Regulation of PI3K/Akt/ER stress was closely involved in the protective effects of GLP-1R (Fig. 8). Overall, these results demonstrate that GLP-1R may be a novel target for the treatment of OA, particularly in patients with diabetes mellitus.

## Materials and methods

### Chemicals and reagents

Liraglutide was obtained from Novo Nordisk (Princeton, NJ, USA). The IL-1β was from Peprotech. Antibodies against GRP78, PDI, IκB-α, p-IκB-α, and collagen II were obtained from Abcam (Cambridge, MA, USA). Antibodies against CHOP, p65, cleaved-caspase3 and specific PI3K inhibitor (LY294002) were obtained from Cell Signaling Technology (Beverly, MA, USA). Antibodies against GLP-1R, MMP3, IL-6, TNF-α, Bax and Bcl-2 were obtained from Santa Cruz Biotechnology (Santa Cruz, CA, USA). Thapsigargin (TG) and other reagents were obtained from Sigma unless noted otherwise.

### Cell isolation and culture

Chondrocytes were extracted using the bilateral knee joints of SD rats (Animal Center of the Chinese Academy of Sciences, Shanghai, China.). All surgical interventions, and postoperative animal care conformed to the Animal Care and Use Committee of Wenzhou Medical University. All rats were housed under specific pathogen-free (SPF) laboratory conditions. Knee-joint cartilage was sectioned into pieces and incubated with 0.25% Trypsin-EDTA for 30 min, and placed in 0.2% type II collagenase for 4 h. After resuspension and filtration, chondrocyts were cultured in DMEM, which contained 10% fetal bovine serum (FBS) and antibiotics (1% penicillin and streptomycin)^60,61^. Morphologically, chondrocytes appear rounded and containing large amounts of aggrecan, a large proteoglycan, and collegan-II, with smaller amounts of other collagens. And Cell fluorescence results of collegan-II showed that most of the cells highly expressed collegan-II (Supplementary Figure S4). Chondrocytes between passage 0 and passage 2 were used for all experiments.

### Cell viability assay

Cell viability was tested using CCK-8 kit (Dojindo, Japan) according to the its protocol. Briefly, cells were plated in 96-well plates. After reaching a confluence of 80–90%, the cells were treated with or without different concentrations of liraglutide (as described) for 2 h, followed by stimulation with or without IL-1β 10 ng/ml for 24 h. Then, CCK-8 dye (10 µL) was added in each well, followed by incubation for 2 h. The absorbance was tested using a 96-well plate reader at 450 nm (Thermo, Rockford, IL, USA).

### TUNEL method

The TUNEL method was used to assess apoptosis in each group of chondrocytes. After fixation with 4% paraformaldehyde (PFA) for 1 h, chondrocytes were incubated with 3% hydrogen peroxide (H_2_O_2_) for 10 min and 0.1% Triton X-100 for 5 min. Then, apoptotic chondrocytes were stained using an in situ Cell Death Detection Kit and subsequently stained with DAPI for 7 min. Images were captured by a Nikon ECLIPSE Ti microscope (Japan).

### Immunofluorescence staining

Immunofluorescence assays were used to assess the protein levels of cleaved-caspase3, p65, MMP3 and collagen II. The chondrocytes were planted on glass coverslips in 6-well plates. After treatment, samples were fixed with 4% PFA for 1 h, and permeabilised by 0.5% Triton X-100 for 10 min. After block with 1% bovine serum albumin (BSA) at 4 °C for 30 min, the primary antibodies were incubated overnight. The next day, samples were incubated with Alexa Fluor 488-conjugated anti-IgG secondary antibodies and Alexa Fluor 647-conjugated anti-IgG for 1 h and stained with DAPI for 7 min. Samples were observed by a Nikon ECLIPSE Ti microscope (Japan).

### Western blot

Total protein of chondrocytes was extracted using RIPA buffer with protease and phosphatase inhibitors. Bicinchoninic acid (BCA) reagents (Thermo, Rockford, IL, USA) were used to measure protein concentrations. Total proteins were loaded onto the SDS-PAGE gels (10–12%) and then transferred to PVDF membranes. Following blocked in 5% BSA for 1 h, the bands were incubated with respective primary antibodies overnight and secondary antibody for 1 h. Finally, the blot signals were visualized using a ChemiDoc™ XRS + Imaging System (Bio-Rad) from at least three independent experiments.

### Small interfering RNA (siRNA)

GLP-1R expression in chondrocytes was silenced by transfection of siRNA. Chondrocytes were transfected with GLP-1R siRNA (GeneChem, Shanghai, China) using lipofectamine 2000 (Life Technologies, Carlsbad, CA, USA) according to the instruction. Six hours after transfection, medium was switched to medium containing 5% FBS for 24 h. Then the chondrocytes were treated as described for further experiments.

### RT-PCR

After treatments, total RNA of cells was isolated using the TRIzol method. After synthesis using the PrimeScript-RT reagent kit from total RNA, the cDNA was amplificated by the PrimeScript-RT reagent kit and SYBR Premix Ex Taq (Sangon). Forward and reverse primer sequences of MMP-3 and collagen-II are showed in Table 1. The expression of each gene was measured by the DDCt method, as mentioned previously^62^.

**Table 1:** Primers of targeted genes

Gene	Forward	Reverse
MMP-3	GCTGTTTTTGAAGAATTTGGGTTC	GCACAGGCAGGAGAAAACGA
Collagen-II	CTCATCCAGGGCTCCAATGA	CCATGGGTGCAATGTCAACA

### OA model

Fifteen male SD rats (200–250 g) were randomly assigned into three groups: Sham, OA, and OA + liraglutide group. Briefly, after anesthesia with chloral hydrate (10%), the right knees of rats were prepared for aseptic surgery. The OA model was cut off anterior cruciate ligament (ACL) transection combined with medial menisci resection on the right knee joint^63^. The rats in the normal group received the same incision without the resection of ACL and meniscus. After surgery, all the rats in the OA + liraglutide group administered at 50 µg/kg/day subcutaneously (s.c.) until the rats were sacrificed. Equal saline injections were administered for the sham group and OA group.

### Histological analysis

Cartilaginous tissue was surgically excised from the right knee joints of the rats and fixed immediately in 4% PFA for 48 h and decalcified for 14 days. Then, the tissues were dehydrated and subsequently embedded in paraffin wax. The tissue sections (5 μm) covering whole joints were cut for HE staining and Safranin O staining to assess cartilage destruction. The images were captured using a light microscope, and the level of cartilage degeneration was measured by the Osteoarthritis Research Society International (OARSI) scores^64^.

### Immunohistochemical analysis

The levels of GLP-1R, cleaved-caspase3 and CHOP were evaluated in each group using immunohistochemical staining. Briefly, the sections were deparaffinised and rehydrated, following blocked with 3% H_2_O_2_ for 10 min and 5% BSA for 30 min. And the tissue sections were incubated with primary antibodies overnight and HRP-conjugated secondary antibody for 1 h, following stained with haematoxylin for 8 min. Images were captured using a light microscope. Images were analysed by Image-Pro Plus software, version 6.0 (Media Cybernetics, Rockville, MD, USA), and the integral absorbance values were used to measure the level of protein. At least three sections from each specimen were used to measure the level of protein.

### Statistical analysis

The results are presented as mean ± SD for each group from at least three independent experiments. Statistical comparisons of the data between each group were conducted using the Graphpad Prism software (one-way analysis of variance (ANOVA) followed by Tukey’s test). *P* values <0.05 were considered statistically significant.

## Electronic supplementary material

Supplementary Figures S1

Supplementary Figures S2

Supplementary Figures S3

Supplementary Figures S4

Supplementary Figure Legends
