# Supplementary figures and images for: Glucagon-like peptide-1 receptor regulates endoplasmic reticulum stress-induced apoptosis and the associated inflammatory response in chondrocytes and the progression of osteoarthritis in rat
Source: Cell Death Dis. 2018 Feb 12;9(2):212. doi: 10.1038/s41419-017-0217-y (PMC5833344; doi:10.1038/s41419-017-0217-y)

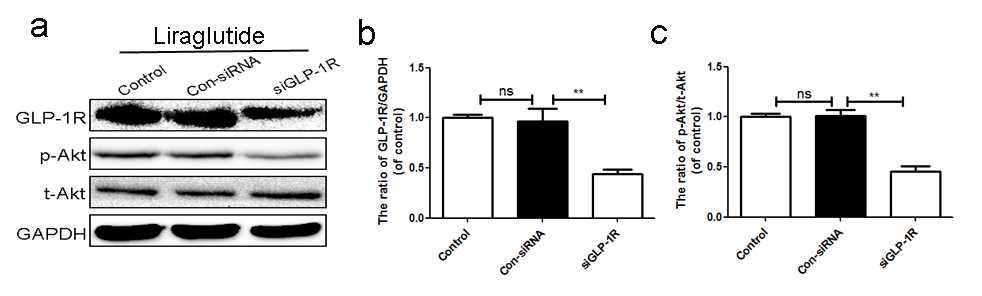

Supplement: Supplementary file 1 — Supplementary Figures S1 [file 41419_2017_217_MOESM1_ESM.tif]

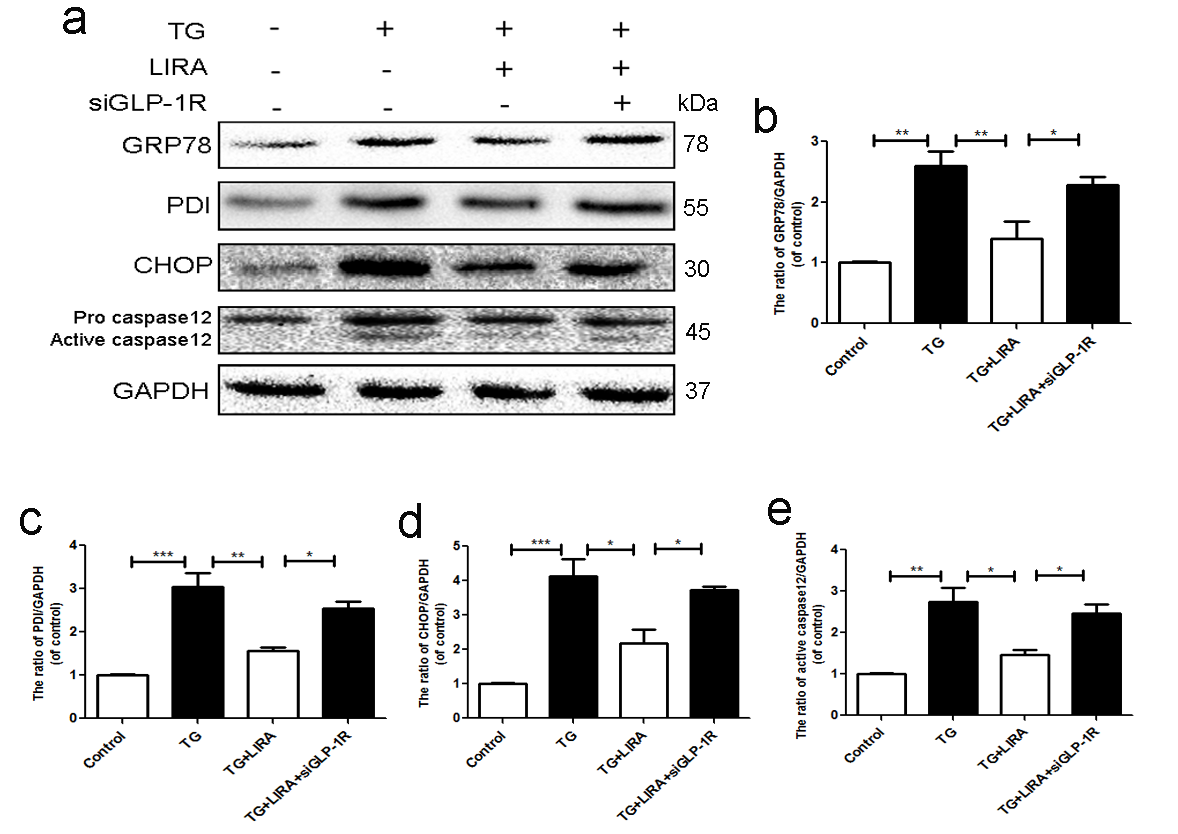

Supplement: Supplementary file 2 — Supplementary Figures S2 [file 41419_2017_217_MOESM2_ESM.tif]

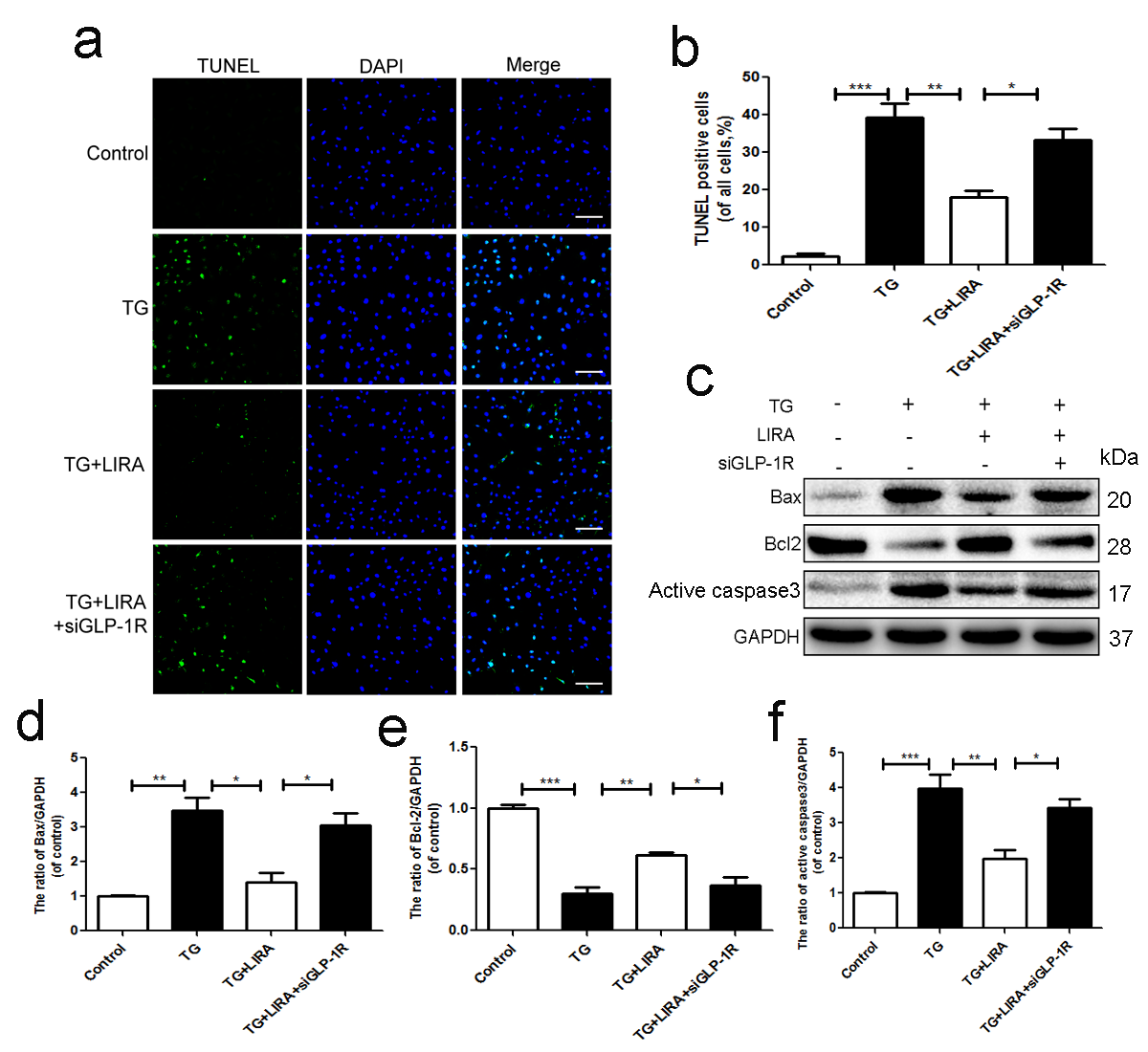

Supplement: Supplementary file 3 — Supplementary Figures S3 [file 41419_2017_217_MOESM3_ESM.tif]

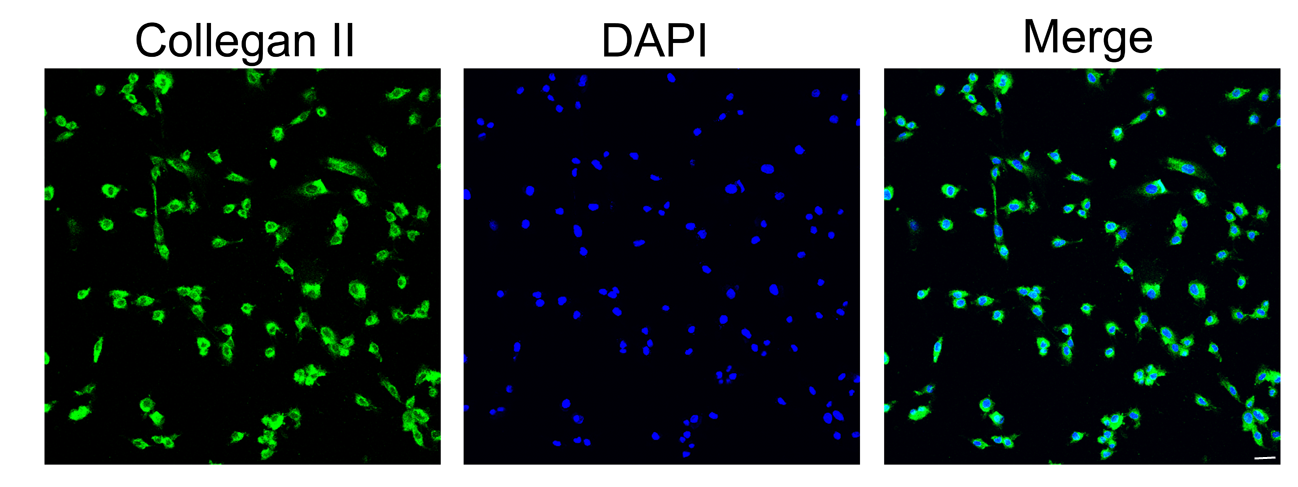

Supplement: Supplementary file 4 — Supplementary Figures S4 [file 41419_2017_217_MOESM4_ESM.tif]
