# Supplementary material for: Glucagon-like peptide-1 receptor regulates endoplasmic reticulum stress-induced apoptosis and the associated inflammatory response in chondrocytes and the progression of osteoarthritis in rat
Source: Cell Death Dis. 2018 Feb 12;9(2):212. doi: 10.1038/s41419-017-0217-y (PMC5833344; doi:10.1038/s41419-017-0217-y)
Supplement: Supplementary file 5 — Supplementary Figure Legends [file 41419_2017_217_MOESM5_ESM.docx]

**Supplementary Figure Legends**

**Supplementary Figures S1** GLP-1R knockdown significantly abolished liraglutide-induced activation of PI3K/Akt signaling in chondrocytes. Chondrocytes were transfected with negative control siRNA (con-siRNA) or GLP-1R siRNA before liraglutide treatment. (a-c) Representative Western blots and quantification data of GLP-1R, p-Akt and Akt in the chondrocytes of each group as described. Data represents the mean ± S.D. Significant differences between the treatment and control groups are indicated as **P<0.01, n=5.

**Supplementary Figures S2** Knockdown of GLP-1R significantly alleviated the effect of liraglutide on TG-induced ER stress in chondrocytes. (a-e) Representative western blots and quantification data of GRP78, PDI, caspase12 and CHOP in each group. Columns represent mean±SD, Significant differences between the treatment and control groups are indicated as *P<0.05, **P<0.01, ***P<0.001, n=5.

**Supplementary Figures S3** Inhibiting GLP-1R abolished the anti-apoptotic effect of liraglutide on TG stimulated-chondrocytes. (a-b) TUNEL assay was used to assess the apoptosis of each group (scale bar: 50μm). (c-f) Representative western blots and quantification data of cleaved caspase 3, Bax and Bcl-2 in each group. Columns represent mean±SD, Significant differences between the treatment and control groups are indicated as *P<0.05, **P<0.01, ***P<0.001, n=5.

**Supplementary Figures S4** Immunofluorescence staining of collegan-II proteins in each group (scale bar: 50μm).
